# Lotus leaf extract and L-carnitine influence different processes during the adipocyte life cycle

**DOI:** 10.1186/1743-7075-7-66

**Published:** 2010-08-05

**Authors:** Ralf Siegner, Stefan Heuser, Ursula Holtzmann, Jörn Söhle, Andreas Schepky, Thomas Raschke, Franz Stäb, Horst Wenck, Marc Winnefeld

**Affiliations:** 1 Research & Development, Research Special Skincare, Beiersdorf AG, Unnastrasse 48, Bf. 520, 20245 Hamburg, Germany

## Abstract

**Background:**

The cellular and molecular mechanisms of adipose tissue biology have been studied extensively over the last two decades. Adipose tissue growth involves both an increase in fat cell size and the formation of mature adipocytes from precursor cells. To investigate how natural substances influence these two processes, we examined the effects of lotus leaf extract (*Nelumbo nucifera*-extract solution obtained from Silab, France) and L-carnitine on human preadipocytes and adipocytes.

**Methods:**

For our *in vitro *studies, we used a lotus leaf extract solution alone or in combination with L-carnitine. Utilizing cultured human preadipocytes, we investigated lotus leaf extract solution-induced inhibition of triglyceride incorporation during adipogenesis and possible effects on cell viability. Studies on human adipocytes were performed aiming to elucidate the efficacy of lotus leaf extract solution to stimulate lipolytic activity. To further characterize lotus leaf extract solution-mediated effects, we determined the expression of the transcription factor adipocyte determination and differentiation factor 1 (ADD1/SREBP-1c) on the RNA- and protein level utilizing qRT-PCR and immunofluorescence analysis. Additionally, the effect of L-carnitine on beta-oxidation was analyzed using human preadipocytes and mature adipocytes. Finally, we investigated additive effects of a combination of lotus leaf extract solution and L-carnitine on triglyceride accumulation during preadipocyte/adipocyte differentiation.

**Results:**

Our data showed that incubation of preadipocytes with lotus leaf extract solution significantly decreased triglyceride accumulation during adipogenesis without affecting cell viability. Compared to controls, adipocytes incubated with lotus leaf extract solution exhibited a significant increase in lipolysis-activity. Moreover, cell populations cultivated in the presence of lotus leaf extract solution showed a decrease in adipocyte differentiation capacity as indicated by a decrease in the ADD1/SREBP-1c signal. Importantly, our results demonstrated that a combination of lotus leaf extract solution and L-carnitine reduced triglyceride accumulation to a greater extent compared to incubation with either substance alone.

**Conclusions:**

Overall, our data demonstrate that a combination of lotus leaf extract and L-carnitine reduced triglyceride accumulation in human (pre)adipocytes by affecting different processes during the adipocyte life cycle. For this reason, this combination might represent a treatment option for obesity-related diseases.

## Background

Throughout industrialized countries, the prevalence of obesity has increased dramatically during the last decades. Since obesity is closely associated with a number of disorders including Type 2 diabetes, hypertension and atherosclerosis, the prevention and treatment of this disease are of major concern [1].

Obesity occurs when the body's energy intake exceeds the body's energy consumption for a prolonged period of time. The degree of obesity is characterized by the volume and number of adipocytes, which is regulated in the so called adipocyte life cycle [2]. Thus, treatments targeting the regulation of adipocyte size and number may provide a therapeutic approach [3,4].

Several plant extracts and their respective bioactive components are well recognized for their potential to exert anti-obesity effects [2]. In this context, we focused on lotus leaf extract, a natural plant extract, obtained from the leaves of *Nelumbo nucifera*. *Nelumbo nucifera *is also known as the sacred lotus and all parts of this plant have been used as traditional medicines in China and India [5]. Lotus leaf extract contains multiple bioactive components such as flavonoids [6], flavonoid glycosides [7] and alkaloids [8]. In obese mice, it has been reported that lotus leaf extract prevented the increase in body weight, inhibited absorption of lipids and carbohydrates, accelerated lipid metabolism and up-regulated energy expenditure, suggesting beneficial effects for the suppression of obesity [9]. Also in mice, it was shown that a 50% ethanol extract prepared from the leaves of *Nelumbo nucifera *stimulated lipolysis in the white adipose tissue [6]. However, to our knowledge conclusive studies investigating effects of lotus leaf extract on triglyceride accumulation or lipolysis-activity in human cells are lacking so far.

Various natural phytochemicals influencing the adipocyte life cycle have been identified. According to Rayalam et al. [2] a selective mono-therapy aiming to prevent or treat obesity has not yet been successfully established. However, the combined use of several natural products, which stimulate different molecular and cellular mechanisms, might represent a more promising approach to treat obesity. Adipose tissue lipolysis leads to the breakdown of triglycerides stored in fat cells and the subsequent release of fatty acids and glycerol. These released fatty acids are transported into the circulatory system and are processed by the liver. Single components upon returning to the adipose tissue can be converted back into adipocyte triglyceride. To prevent this process, stimulation of beta-oxidation and, thus, the removal of fatty acids (by creating energy) might help to decrease fat stores more efficiently [10-12].

To determine to which extent lotus leaf extract influences adipogenesis and induces lipolysis-activity, we investigated the effects of a lotus leaf extract solution on human preadipocytes and adipocytes. Since L-carnitine functions as a stimulant for the oxidation of fatty acids, we additionally determined the possibility of combined effects of lotus leaf extract and L-carnitine in human (pre)adipocytes.

## Methods

### Test substances

#### Lotus leaf extract solution

For our studies, a liquid leaf extract of *Nelumbo nucifera *(Pro-Sveltyl OP^®^; Lot 621021 and 621031; Silab, Brive, France) designated as 'lotus leaf extract solution' was used. This liquid extract solution is obtained from *Nelumbo nucifera *leafs and comprises of a dry matter content of approximately 6.4 mg/ml in a 50% aqueous butylene glycol solution. To control for possible effects induced by the butylene glycol solution, an aqueous 50% butylene glycol solution in the respective concentration was used as control.

#### L-carnitine

The second substance used for our experiments was L-carnitine (L-Carnipure crystalline; Lot 00002; Lonza, Basel, Switzerland). For experiments on beta-oxidation, L-carnitine was dissolved directly in the respective cell culture medium. In fat accumulation experiments, L-carnitine was dissolved in cell culture medium in the presence of 0.25% butylene glycol. This experiment allowed us to compare the L-carnitine-induced effects with the actions mediated by the combination of L-carnitine and 0.5% lotus leaf extract solution which had a final concentration of 0.25% butylene glycol. The respective control was treated accordingly.

#### Differentiation of preadipocytes into adipocytes

Subcutaneous human preadipocytes isolated from the abdomen, thigh and waist of different healthy subjects (LOT L012202, L060403T, L052206) were obtained from Zen-bio Inc. (Research Triangle Park, NC). Human adipose derived stem cells (hADSC, LOT 7F3400) were obtained from Cambrex (Verviers, Belgium). Cells were cultured as previously described [13]. Briefly, cells were incubated in basal growth medium (PGM, Cambrex, Verviers, Belgium) containing 10% fetal calf serum (FCS), 2 mM L-glutamine, 100 U/ml penicillin and 100 μg/ml streptomycin (Cambrex, Verviers, Belgium) for 7 days at 37°C and 5% CO_2 _with medium change taking place at day 1 and 4 after seeding. Following incubation, cells were seeded into 96-well plates (1 × 10^4 ^per well) or 6-well plates (3 × 10^5 ^per well), and after incubation overnight the differentiation into adipocytes was initiated by addition of 10 μg/ml insulin, 1 μM dexamethasone, 200 μM indomethacin and 500 μM isobutylmethylxanthine (Cambrex, Verviers, Belgium) to the medium. Culture medium containing these ingredients is designated as 'differentiation medium'. Cells cultured for 7 days or more post induction of differentiation, are referred to as adipocytes, while cells cultured without the differentiation stimulus are termed preadipocytes. For our studies we used cells up to the third passage.

#### Determination of cell viability

A viability assay determining the endogenous esterase activity was used as previously described [14] to evaluate possible cytotoxic effects of lotus leaf extract solution. Briefly, preadipocytes were cultured for 7 days in 'differentiation medium' containing 0.5%, 1% lotus leaf extract solution or the respective control solution. Subsequently, cells were washed with 1× Dulbecco's Phosphate Buffered Saline (DPBS) (Cambrex, Verviers, Belgium) and incubated for 20 min in 100 μl fluorescein diacetate (FDA; Sigma, Taufkirchen, Germany) solution (15 μg/ml FDA in 1× DPBS). Fluorescence was then determined (excitation at 491 nm/emission at 517 nm) using the 96-well plate reader Safire 1 (Tecan, Crailsheim, Germany).

#### Determination of triglyceride accumulation

For experiments, preadipocytes were cultivated for 7 days in 'differentiation medium' supplemented with 0.5% or 1% lotus leaf extract solution, 0.01% L-carnitine, a combination of 0.01% L-carnitine and 0.5% lotus leaf extract solution or the respective control solutions.

The accumulation of triglycerides during differentiation was determined on day 7 using an AdipoRed Assay (Cambrex, Verviers, Belgium) according to the manufacturer's instructions. Fluorescence was detected (excitation at 485 nm/emission at 572 nm) and quantified in a 96-well plate reader Safire 1 (Tecan, Crailsheim, Germany).

For additional microscopic analysis, cells were incubated with AdipoRed reagent for 10 min at room temperature. Samples were analyzed by fluorescence microscopy using an Olympus IX71 microscope (Hamburg, Germany).

#### Determination of glycerol release

Subcutaneous human preadipocytes or hADSCs were seeded in cell culture flasks (9 × 10^3 ^cells/cm^2^) and cultured in Dulbecco's modified Eagle Medium-F12 (Gibco/BRL, Eggenstein, Germany) containing 10% FCS, 0.5% gentamycin (Gibco/BRL, Eggenstein, Germany), 33 μM biotin and 17 μM D-pantothenate (Sigma, Taufkirchen, Germany) at 37°C and 5% CO_2_. After 7 days, cells were transferred into 96-well plates (10^5 ^cells/well). One day later, the medium was replaced with fresh medium containing 10% FCS, 0.5% gentamycin, 33 μM biotin, 17 μM D-pantothenate, 66 nM insulin, 1 nM T3, 100 nM hydrocortisol, 0.1 μg/ml apo-Transferrin and 1 μg/ml ciglitazone (all obtained from Sigma, Taufkirchen, Germany). After 3 days, the medium was substituted for medium of the same composition but lacking ciglitazone. Three days later, this medium was replaced by 'differentiation medium' and, cells were cultivated for 2 additional weeks.

Prior to incubation with test substances, the differentiated cells were cultured for one week in Dulbecco's modified Eagle Medium low Glucose (Cambrex, Verviers, Belgium) supplemented with 1% Bovine Albumin Fraction V, 100 U/ml penicillin, 100 μg/ml streptomycin and 1× Glutamax (all obtained from Gibco/BRL, Eggenstein, Germany). This culture medium is designated as 'maintenance medium'. Glycerol release was analyzed 28 days post induction of differentiation.

For experiments, cells were incubated in 200 μl 'maintenance medium' containing either 0.5% or 1% lotus leaf extract solution or control solution for 24 h at 37°C and 5% CO_2_. Free glycerol reagent and standard solution (Sigma, Taufkirchen, Germany, standard dilution: 125 to 1.95 μg/ml (1:1 mix ratio steps)) were used according to the manufacturer's instructions.

For measurement, 100 μl cell supernatant were obtained from each well and mixed with 100 μl free glycerol reagent using a 96-well plate. After 15 min of incubation at room temperature in the dark, absorption was measured in a Spectra MAX 96-well plate reader (Molecular Devices, Union City, CA) at 540 nm.

#### Quantification of ADD1/SREBP-1c gene expression

Subcutaneous human preadipocytes were cultured as described above. For experiments, cells were cultivated for 3, 6 and 9 days in 'differentiation medium' with or without 1% lotus leaf extract solution. Cells were harvested on day 3, 6 and 9 after induction of differentiation and homogenized in TRIzol^® ^(Invitrogen, Karlsruhe, Germany) following the manufacturer's protocol. After reverse transcription, samples were analyzed for the adipocyte determination and differentiation factor 1 (ADD1/SREBP-1c) by Real-Time TaqMan^®^-PCR using the 7900HT Fast-Real-Time PCR System (Applied Biosystems, Darmstadt, Germany).

FAM labelled primers for qRT-PCR (Applied Biosystems, Forster City, CA) were as follows: Inventoried TaqMan Assays for the internal control glyceraldehyde-3-phosphate dehydrogenase (GAPDH; Hs99999905_m1) and for the target RNA ADD1/SREBP-1c (Hs01088691_m1). TaqMan^® ^Fast Universal PCR Master Mix (Applied Biosystems, Forster City, CA) was used and the PCR was performed as recommended by the supplier. Real-time PCR data were analyzed using the Sequence detector version 2.3 software supplied with the 7900HT Fast-Real-Time PCR System (Applied Biosystems, Darmstadt, Germany). Quantification was achieved using the 2^-ΔΔCt ^method which calculates the relative changes in gene expression of the target normalized to an endogenous reference (GAPDH).

#### Immunofluorescence microscopic analysis

For immunofluorescence analysis, preadipocyte populations were grown on coverslips and incubated in 'differentiation medium' with or without 1% lotus leaf extract solution for 9 days as described above. Next, cells were fixed with 4% formaldehyde solution for 30 min at room temperature, washed with phosphate buffered saline (PBS) and permeabilized with 0.2% Triton X-100. After successive washing with PBS, fixed cells were pre-treated with PBS containing 10% donkey-serum for 30 min. Cells were then incubated for 1 h with primary antibodies directed against ADD1/SREBP-1c (sc 8984; Santa Cruz, Heidelberg, Germany). Coverslips were successively rinsed three times with PBS, and then incubated for 1 h with a secondary antibody labeled with Cy3. Cell nuclei were stained using Hoechst 33342 (1 μg/ml; Invitrogen, Karlsruhe, Germany). Results were determined using the Fluorescence microscope IX71 in combination with the software cell^F v. 2.4 (Olympus, Hamburg, Germany).

#### Determination of beta-oxidation

For determination of beta-oxidation, subcutaneous human preadipocytes were cultured according to the manufacturer's instructions as described above. Preadipocytes (300,000 cells/dish) were seeded into sterile 3.3 cm Nunclon Petri dishes (Nunc, Roskilde, Denmark) in PGM. The next day, beta-oxidation was measured as described below.

With respect to adipocyte populations, differentiation was induced by adding 10 μg/ml insulin, 1 μM dexamethasone, 200 μM indomethacin and 500 μM isobutylmethylxanthine (Cambrex, Verviers, Belgium) to the medium. Cells were cultured for 2 weeks with one medium change taking place after one week of culture. Prior to determination of beta-oxidation, adipocytes were incubated for 7 days in 'maintenance medium'.

For experiments, adipocytes were cultivated in 'maintenance medium' with and without 0.01%, 0.025% and 0.1% L-carnitine while preadipocytes were incubated with 0.1% L-carnitine, 0.5% or 1% lotus leaf extract solution solved in PGM. The medium was additionally supplemented with 8.8 μM ^14^C-labeled palmitic acid (Amersham Buchler GmbH, Braunschweig, Germany). Each dish was incubated for 18 h at 37°C in a sealed 125 ml Nalgene sterile plastic container (Nunc, Roskilde, Denmark) equipped with a rubber septum in its lid. In addition, the container contained a 2 ml reaction tube located in a purpose-built reck. Following incubation, 1 ml benzethonium hydroxide solution (Sigma-Aldrich, Steinheim, Germany) was injected as a ^14^CO_2 _scavenger into the reaction tube via the rubber septum using a syringe with a cannula. Cells were then lysed and ^14^CO_2 _was driven out by the injection of 600 μL of 7% trichloroacetic acid into the cell supernatant via the rubber septum. Following an incubation of 2 h at room temperature under gentle shaking, the sealed system was opened and the ^14^CO_2 _scavenger solution was transferred into a scintillator cup. Two mL of Ultima Gold scintillator liquid (Perkin Elmer, Boston, MA) were added and radioactivity was determined using a Beckman LS6500 liquid scintillation counter device (Beckman Coulter, Brea, CA). The amount of radioactive ^14^CO_2 _served as a measure for the quantity of beta-oxidation. Counts of control samples cultivated without L-carnitine or lotus leaf extract solution were set as 100%.

### Statistical analysis

A significance level of 0.05 (alpha) was chosen for statistical analysis, based on two-sided hypothesis testing.

The following analysis was performed:

Check of normal distribution by means of Shapiro-Wilk's test.

Comparison versus control by means of repeated measures ANOVA with treatment as classification variable.

Comparison between experiments by means of ANCOVA with treatment as classification variable and control as covariable.

Where required, post-hoc pairwise comparison by means of generalized Tukey test.

Software used: SAS software package for Windows V9.1.3.

## Results

### Lotus leaf extract solution decreased triglyceride accumulation during adipogenesis

**Figure 1:**
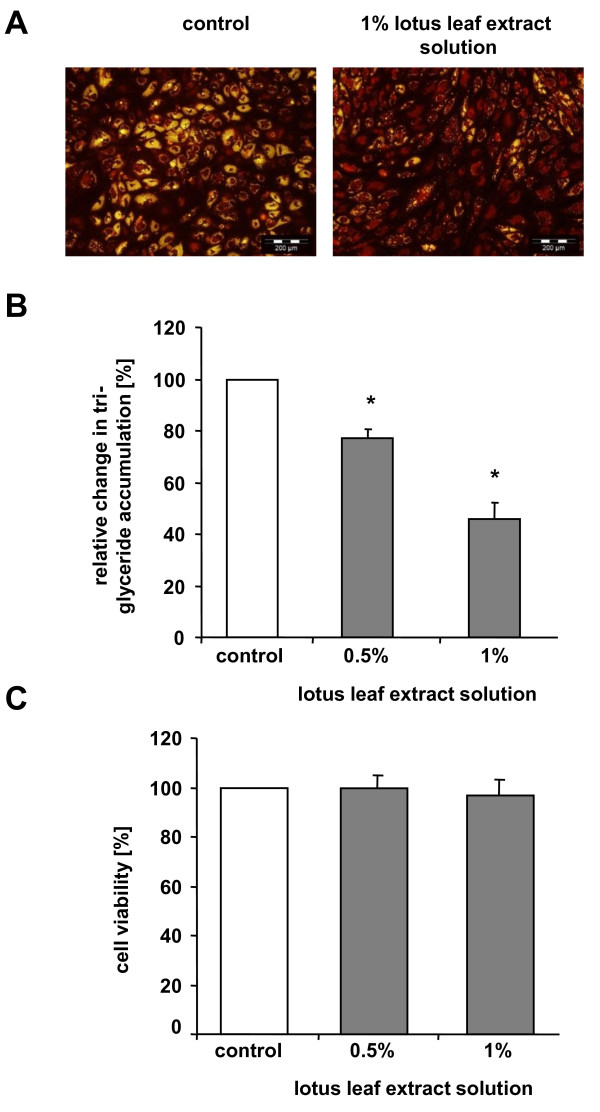
**Lotus leaf extract solution decreased triglyceride accumulation during preadipocyte/adipocyte differentiation**. (A) Images after triglyceride staining (yellow) from cell populations incubated with and without 1% lotus leaf extract solution. Scale bar: 200 μm. (B) Triglyceride accumulation of maturing human adipocytes incubated with 0.5% or 1% (n = 12, each) lotus leaf extract solution relative to untreated control cells (n = 12) set as 100%. Results are depicted as mean ± SD. (C) Cell viability of maturing adipocytes cultivated in 'differentiation medium' with 0.5% or 1% lotus leaf extract solution (n = 12, each) compared to untreated control cells set as 100%. Results are depicted as mean ± SD. Significant differences are marked with an asterisk (* for p < 0.0001 against control).

To investigate effects of lotus leaf extract solution on triglyceride accumulation during human preadipocyte/adipocyte differentiation, cells were cultured in 'differentiation medium' for 7 days in the absence or presence of lotus leaf extract solution. As displayed in Figure 1A, the majority of control cells showed an accumulation of triglycerides in the lipid droplets. In contrast, most cells incubated with 1% lotus leaf extract solution did not accumulate triglycerides as indicated by the absence of yellow staining.

To quantify these results, the total amount of triglyceride accumulation during (pre)adipocyte differentiation was determined (Figure 1B). Cells were cultured in the absence or presence of 0.5% (n = 12) or 1% (n = 12) lotus leaf extract solution. The results showed that lotus leaf extract solution decreased triglyceride accumulation in a dose-dependent manner. Cells treated with 'differentiation medium' containing 0.5% lotus leaf extract solution significantly (p < 0.0001) decreased triglyceride levels to 77 (± 3.4)% compared to control cells (set as 100%) whereas incubation with 1% lotus leaf extract solution significantly reduced (p < 0.0001) triglyceride accumulation to 46 (± 5.9)%.

In addition, a viability assay was used to determine any possible adverse effects of 0.5% (n = 12) or 1% (n = 12) lotus leaf extract solution. As illustrated in Figure 1C, control cells (set as 100%) and maturing adipocytes cultivated with 0.5% (100 ± 4.9%) or 1% lotus leaf extract solution (97 ± 6%) displayed comparable esterase activities indicating that the viability of cultured cells was not affected by incubation with lotus leaf extract solutions.

### Lotus leaf extract solution stimulated lipolysis-activity in differentiated adipocytes

**Figure 2:**
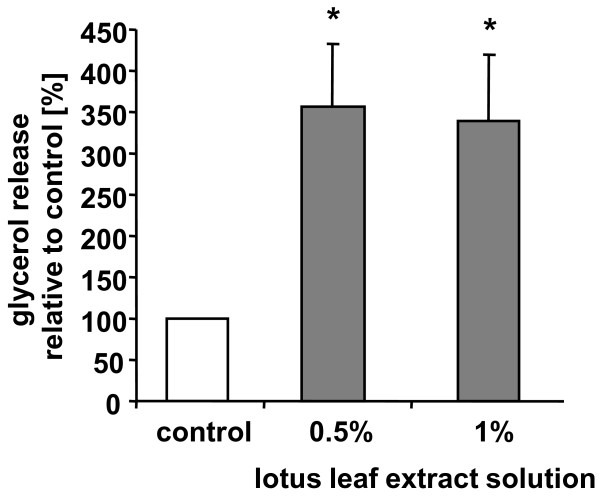
**Increase in glycerol release from differentiated adipocytes after incubation with lotus leaf extract solution**. Glycerol release from differentiated human adipocytes after treatment with 0.5 or 1% lotus leaf extract solution (n = 18) compared to untreated control cells set as 100%. Results are depicted as mean ± SD. Significant differences are marked with an asterisk (* for p < 0.0001 against control).

To address the question whether lotus leaf extract solution also stimulates lipolysis-activity by degrading triglycerides into free fatty acids and glycerol, we determined glycerol release from cell populations (n = 18) cultivated in the presence or absence of lotus leaf extract solution. As our studies showed (Figure 2), treatment of adipocyte cultures with 0.5% lotus leaf extract solution significantly (p < 0.0001) increased the content of free glycerol to 356 (± 76)% compared to control cells (set as 100%). Likewise, cultivation of cells with 1% lotus leaf extract solution induced a significant (p < 0.0001) release of free glycerol to 340 (± 80)% compared to control cells.

### Effects of lotus leaf extract solution on ADD1/SREBP-1c mRNA- and protein levels

**Figure 3:**
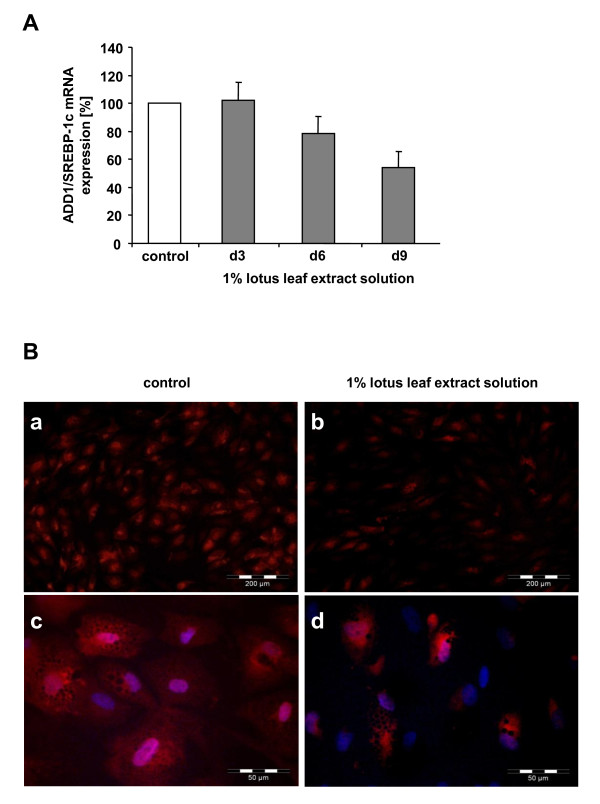
**Effect of lotus leaf extract solution on ADD1/SREBP-1c expression during adipogenesis**. (A) ADD1/SREBP-1c gene expression in differentiating preadipocytes after incubation with 1% lotus leaf extract solution compared to control cells set as 100%. Expression of ADD1/SREBP-1c is normalized to GAPDH. Three independent experiments were prepared both for control and incubation with lotus leaf extract solution (n = 3). Results are depicted as mean ± SD. (B) Human preadipocyte populations were cultured in 'differentiation medium' without (a, c) or with (b, d) 1% lotus leaf extract solution for 9 days. Immunofluorescence staining of ADD1/SREBP-1c (red) and of DNA (Hoechst-33342 (blue)) was performed. a and b: Scale bar: 200 μm; c and d: Scale bar: 50 μm.

To gain insight into the molecular events associated with the above described inhibition of triglyceride accumulation, cells were cultivated in the presence or absence of 1% lotus leaf extract solution. Three, 6 and 9 days after initiation of differentiation, gene expression of the essential adipogenic transcription factor ADD1/SREBP-1c was determined relative to control cells by quantitative RT-PCR (Figure 3A; n = 3). Compared to control cells (set as 100%), pre(adipocytes) incubated with 1% lotus leaf extract solution displayed a decrease in ADD1/SREBP-1c mRNA levels over time.

To investigate if the effects of lotus leaf extract solution on ADD1/SREBP-1c gene expression translate to the protein level, we incubated cells with 1% lotus leaf extract solution and determined the expression of ADD1/SREBP-1c using immunofluorescence analysis. In agreement with our previous findings, cells incubated with lotus leaf extract solution (Figure 3B b and 3B d) displayed only a weak ADD1/SREBP-1c signal compared to the control cells (Figure 3B a and 3B c). Notably, the number of nuclei, stained with Hoechst 33342, was comparable in all investigated areas.

In summary, the results indicated that lotus leaf extract solution affects adipogenesis.

### Determination of effects of L-carnitine on beta-oxidation

**Figure 4:**
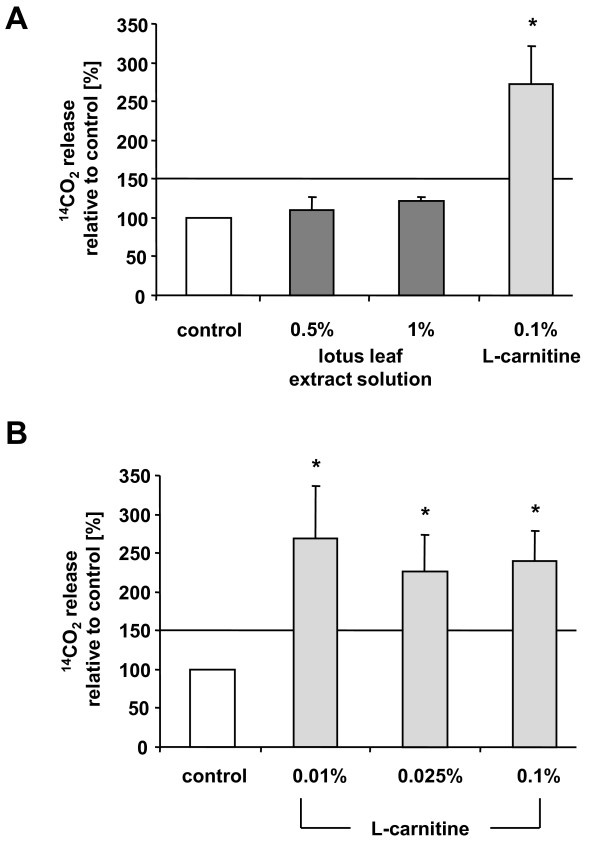
**L-carnitine stimulated beta-oxidation in preadipocytes and differentiated adipocytes**. (A) Preadipocytes were incubated with 0.1% L-carnitine, 0.5% and 1% lotus leaf extract solution or left untreated as control for 18 h (n = 7). (B) Differentiated adipocytes were cultivated with 0.01%, 0.025% or 0.1% L-carnitine and untreated cells served as control (n = 14). ^14^CO_2 _counts emitted by control samples were set as 100%. The threshold for relevant differences was set at 150%. Results are depicted as mean ± SD. Significant differences are marked with an asterisk (* for p < 0.0001 against control).

To characterize a possible effect of L-carnitine and lotus leaf extract solution on beta-oxidation in human preadipocytes (n = 7) and differentiated adipocytes (n = 14), cells were incubated with L-carnitine, lotus leaf extract solution or without as described in the figure legend. As depicted in Figure 4A and 4B, the release of radioactively-labeled CO_2 _was significantly (p < 0.0001) augmented in preadipocyte populations cultivated in the presence of 0.1% L-carnitine (272 ± 48%) compared to controls (set as 100%). Similar results were observed in adipocyte cell cultures (240 ± 39%). In addition, in adipocyte populations an increase in radioactively-labeled CO_2 _was also observed when 0.025% (227 ± 46%) and 0.01% (269 ± 67%) L-carnitine was used, indicating that even lower concentrations of L-carnitine affect beta-oxidation. In contrast, preadipocyte incubation with 0.5% or 1% lotus leaf extract solution did not sufficiently affect the release of radioactively-labeled CO_2_.

### Additive effects of lotus leaf extract solution and L-carnitine on triglyceride accumulation

**Figure 5:**
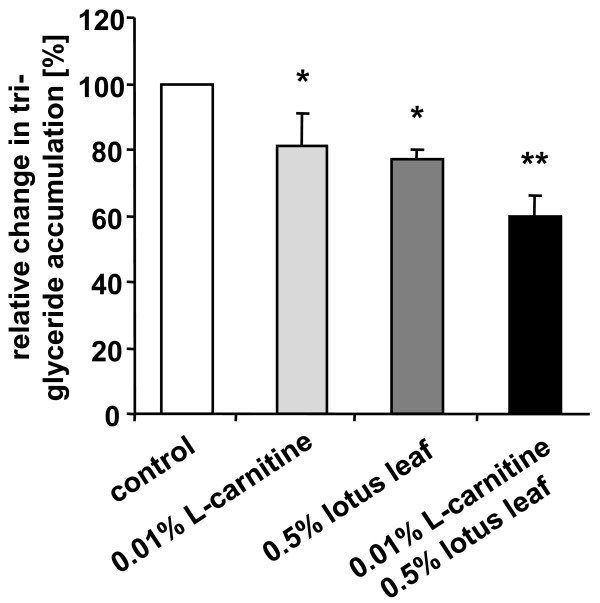
**Additive effect of lotus leaf extract solution and L-carnitine on triglyceride accumulation during preadipocyte/adipocyte differentiation**. Treatment of preadipocytes with 0.01% L-carnitine (n = 10), 0.5% lotus leaf extract solution (n = 12) or a combination of 0.01% L-carnitine and 0.5% lotus leaf extract solution (n = 12) on triglyceride accumulation during preadipocyte/adipocyte differentiation. Triglyceride levels of the respective control samples were set as 100%. Results are depicted as mean ± SD. Significant differences are marked with an asterisk (* for p ≤ 0.0001 against control; ** for p ≤ 0.0001 against control and all other treatments).

Our previous results showed that L-carnitine stimulated beta-oxidation in preadipocytes as well as in mature adipocytes. Therefore, we investigated if a combined treatment of cells with L-carnitine and lotus leaf extract solution might result in a pronounced reduction of triglyceride accumulation during preadipocyte/adipocyte differentiation. As our data showed (Figure 5), compared to the respective control (set as 100%), incubation of cells with 0.01% L-carnitine (n = 10) or 0.5% lotus leaf extract solution (n = 12) alone induced a significant decrease in triglyceride accumulation to 81 (± 9.6)% (p = 0.0001) and 77 (± 3.4)% (p < 0.0001), respectively. However, supplementation of the cell culture medium with a combination of 0.01% L-carnitine and 0.5% lotus leaf extract solution (n = 12) reduced triglyceride levels significantly (p < 0.0001) to 60 (± 5.7)% compared to control cells (set as 100%). In addition, triglyceride levels of cells incubated with L-carnitine and lotus leaf extract solution were significantly (p < 0.0001, each) lower than triglyceride levels of cell populations which were cultivated in the presence of only one of the two actives.

## Discussion

The simultaneous regulation of different mechanisms involved in fat metabolism utilizing multiple natural compounds might be an appropriate approach to increase anti-obesity treatment efficiently. To put this approach into practice, we aimed to identify active ingredients that stimulate different processes. In a second step, we tested if a combination of these ingredients is able to cause a sustained decrease in fat stores.

To address the first part, we primarily studied the effects of lotus leaf extract solution on triglyceride accumulation and lipolysis-activity in human (pre)adipocytes.

As our data demonstrate, incubation with 1% lotus leaf extract solution decreased the number and content of lipid droplets during preadipocyte differentiation compared with control cells. In addition, both 0.5% and 1% lotus leaf extract solution significantly reduced triglyceride incorporation in human preadipocytes during adipogenesis in a dose-dependent manner. Importantly, this significant reduction in triglyceride accumulation was not due to toxic side effects exerted by the lotus leaf extract solution.

To investigate if the observed attenuation in triglyceride incorporation during adipogenesis might be due to the ability of lotus leaf extract solution to increase the conversion of triglycerides to fatty acids and glycerol, we measured the glycerol content in the supernatants of differentiated adipocyte cultures. Interestingly, our results show that lotus leaf extract solution significantly augmented lipolysis-activity.

In the literature it has been reported that a number of natural compounds inhibit adipogenesis on a molecular level [2]. The complex sequence of preadipocyte differentiation is initially triggered by transcription factor-activated signaling pathways [15]. Three classes of transcription factors are directly involved in adipogenesis: the peroxisome proliferator-activated receptor γ (PPARγ), the CCAAT/enhancer binding proteins (C/EBPα, C/EBPß and C/EBPδ) and the adipocyte determination and differentiation factor 1 (ADD1/SREBP-1c) [3]. ADD1/SREBP-1c is highly expressed in the liver and in adipose tissue and plays a crucial part in adipocyte differentiation [16]. ADD1/SREBP-1c mRNA levels are augmented in determined preadipocytes and to a greater extent increased during the differentiation process [16,17]. In addition to its role in adipogenesis, ADD1/SREBP-1c has been associated with the regulation of genes linked to cholesterol metabolism. In this context, ADD1/SREBP-1c has been termed sterol regulatory element binding protein 1c (SREBP-1c) [18].

To study if incubation with lotus leaf extract solution is associated with adipocyte differentiation, we investigated potential alterations in the mRNA- and protein levels of ADD1/SREBP-1c. Our results illustrate that differentiated adipocytes displayed an augmented ADD1/SREBP-1c mRNA and protein expression. Interestingly, cells incubated in the presence of 1% lotus leaf extract solution showed a decreased ADD1/SREBP-1c mRNA expression. In addition, these cells exhibited only a weak ADD1/SREBP-1c signal as determined by immunofluorescence analysis indicating that cultivation in the presence of lotus leaf extract solution affects adipocyte differentiation.

According to our starting idea whether triglyceride accumulation can be reduced to an even greater extent by regulating another important process in fat metabolism, we investigated the impact of L-carnitine (alone or in combination with lotus leaf extract solution) on human primary preadipocytes/adipocytes.

L-carnitine (β-hydroxy-γ-trimethylaminobutyric acid), a small, water-soluble, quaternary amine is known to play a role in mammalian lipid catabolism and maintenance of the body's energy metabolism. In humans, its homoeostasis is maintained by endogenous synthesis performed by the kidney, liver and brain, absorption from dietary sources and efficient tubular re-absorption by the kidney. Its daily requirements are mainly satisfied by diet. Major sources of L-carnitine in the human diet are meat, fish and dairy products [19]. L-carnitine represents an essential cofactor for long-chain fatty acid oxidation translocating long chain fatty acids across the mitochondrial membranes into the mitochondrial matrix, where beta-oxidation takes place. Through this mechanism, it supports the degradation of fatty acids and the concomitant production of biological energy in the form of ATP.

The role of L-carnitine supplementation in obesity treatment is discussed controversially in the scientific literature. In healthy adults without L-carnitine deficiency, fatty acid oxidation increased after L-carnitine supplementation (3 g/day) for 10 days [20]. In addition, in rats fed a high fat diet, L-carnitine supplementation significantly decreased food intake, whole body weight and adipose tissue accumulation [21]. On the other hand, there are data suggesting that L-carnitine supplementation is not effective in inducing changes in body composition or weight reduction [22].

At the molecular level an anti-obesity effect of L-carnitine is suggested by Lee and co-workers. They demonstrated in 3T3-L1 cells that L-carnitine stimulates lipolysis-activity and increases the expression of genes involved in beta-oxidation [23]. Although the mouse 3T3-L1 cell line represents a well-established model system to study fat metabolism [24], known species-related differences in adipose tissue biology have to be taken into account. For this reason, we utilized primary cultured human cells throughout our experiments as these cells represent a better *in vitro *model for humans. In line with the study mentioned above [23], we detected a significantly augmented beta-oxidation in human preadipocytes and differentiated adipocytes after incubation with L-carnitine compared to control cells. However, a stimulation of lipolysis-activity by L-carnitine was not observed (data not shown). Furthermore, an increase in beta-oxidation was not induced by lotus leaf extract solution, showing that both ingredients act on different processes during the adipocyte life cycle.

Murosaki et al. [11] demonstrated combined effects of L-carnitine, soy isoflavones, caffeine and arginine on triglyceride accumulation, lipolysis-activity and fatty acid oxidation using 3T3-L1 or HepG2 cells. Moreover, the combination of these compounds significantly suppressed lipogenesis in the liver when KK mice were food-deprived for 48 h and subsequently re-fed a fat free diet. In addition, body weight, adipose tissue weight, and triglyceride levels in the plasma and liver were reduced in obese KK mice given a low-fat diet. In a rat model with high fat diet-induced obesity a mixture composed of L-carnitine, *G. cambogia *extract and soy peptide attenuated visceral fat accumulation and improved dyslipidemia [25]. These reports support the notion that L-carnitine used in combination with other natural compounds of different origin can induce beneficial effects in obesity treatment.

Based on the findings of Murosaki and co-workers [11], we studied the combined effects of lotus leaf extract solution and L-carnitine on triglyceride accumulation during human preadipocyte/adipocyte differentiation. In line with our previous results, incubation of cells with 0.01% L-carnitine or 0.5% lotus leaf extract solution alone induced a significant decrease in triglyceride accumulation. However, compared with control cells, supplementation of cell culture medium with a combination of L-carnitine and lotus leaf extract solution resulted in an even greater reduction in triglyceride levels, clearly pointing to an additive effect induced by these two compounds. Nevertheless, the molecular pathway(s) leading to the described additive effect will have to be identified in future studies.

Although final confirmation is still lacking, it might be speculated that the increased (lotus leaf extract solution-induced) lipolysis-activity in combination with the augmented (L-carnitine-induced) degradation of fatty acids not only leads to an increase in triglyceride degradation but also results in the elimination of triglycerides from the metabolic system. With respect to anti-obesity treatments, the last point is crucial, since accumulation of mobilized fatty acids in other tissues such as muscle or liver needs to be prevented.

## Conclusion

A wide range of health problems is related to an improper fat storage and release. Due to the increase in obesity-related disorders, cellular and molecular processes underlying fat metabolism have been investigated intensely in recent years.

In this context, we aimed to test a combination of natural compounds influencing different processes during the adipocyte life cycle to achieve synergistic and/or additive effects on cellular fat reduction. Overall, our data demonstrate that single treatment of primary human (pre)-adipocytes with lotus leaf extract solution modulated lipolysis-activity and also decreased adipogenesis. Additionally, L-carnitine augmented beta-oxidation in primary human adipocytes. Importantly, the combined treatment utilizing lotus leaf extract solution and L-carnitine led to a significant reduction in triglyceride accumulation compared to the single use of these natural sources. By simultaneously stimulating different pathways associated with fat storage a better potential for the treatment and prevention of obesity might be achieved.

## Competing interests

The authors declare that they have no competing interests.

## Authors' contributions

RS performed the experiments concerning beta-oxidation and qRT-PCR. Moreover, he assisted with interpretation of the results and helped draft the manuscript. RS and UH analyzed the triglyceride accumulation and viability during adipocyte differentiation. JS analyzed the lipolytic activity of differentiated adipocytes and performed the immunofluorescence experiments. SH, TR AS, HW and FS assisted with interpretation of the results. MW supervised the analyses and helped to draft the manuscript. All authors read and approved the final manuscript.
